# Tuberculous Chronic Palmar Bursitis and Flexor Tenosynovitis of the Wrist: A Case Report

**DOI:** 10.7759/cureus.82652

**Published:** 2025-04-20

**Authors:** Shweta Kataria, Saurabh Gangwar, Sravan K Marupaka, Abhijeet Ingle

**Affiliations:** 1 Department of Radiology, University of North Carolina at Chapel Hill, Chapel Hill, USA; 2 Department of Radiology, Rajshree Medical Research Institute, Bareilly, IND; 3 Department of Radiology, Deccan College of Medical Sciences, Hyderabad, IND; 4 Department of Pathology, Yashoda Hospitals, Hyderabad, IND

**Keywords:** chronic flexor tenosynovitis, compound palmar ganglion, horseshoe abscess, musculoskeletal tb, palmar bursitis, rice bodies

## Abstract

Palmar bursitis and tenosynovitis of the flexor tendon sheath around the wrist is a rare manifestation of musculoskeletal tuberculosis. It has an indolent course and diagnosis is often delayed especially when it is an isolated finding. Timely diagnosis can prevent complications like bony involvement, tendon rupture and neuropathy. Here, we report a case of a 42-year-old male patient who presented with gradually progressive painful swelling of the left palm, wrist and distal forearm. Ultrasound and MRI revealed synovial thickening and distension of the palmar bursae and flexor tendon sheath with fluid and rice bodies. Open excisional biopsy was done, and thickened synovium and rice bodies were removed. Histopathology showed epithelioid granulomas with Langhans type giant cells and caseation necrosis. Anti-tubercular therapy was administered, and on follow-up, there was no recurrence of symptoms.

## Introduction

Musculoskeletal tuberculosis accounts for 1%-5% of total tuberculosis cases, and spine is the most common site of involvement [1]. Primary tuberculous pyomyositis, bursitis, and tenosynovitis are rare and account for about 1% of skeletal tuberculosis cases [2]. Tuberculous tenosynovitis commonly involves the flexor compartment of the wrist [2]. It is also referred to as compound palmar ganglion and involves palmar bursae, manifesting as an hourglass-shaped fluctuant swelling proximal and distal to the flexor retinaculum. The inflamed synovium is thickened and the bursa is distended with fluid and rice bodies. Early diagnosis is essential to prevent complications and disability. MRI plays crucial role in diagnosis due to characteristic imaging features and determining the extent of involvement. In this report, we have discussed a case of chronic palmar bursitis and flexor tenosynovitis of the wrist with rice body formation. Histopathology was suggestive of tuberculous etiology. To the best of our knowledge, there has been no reported case with such long duration of symptoms without bone involvement. Previous reports have focused mainly on the clinical aspect of the disease. We have emphasized on the imaging features on ultrasound and MRI and histopathology findings, with a brief discussion of anatomy.

## Case presentation

A 42-year-old man presented to our hospital with a mildly painful swelling of the left wrist and hand that had been gradually increasing in size for the past three years. There was no history of any other joint involvement. The patient had a remote history of trauma to the left distal forearm. On examination, there was a slightly tender swelling on the volar side of the left distal forearm and hand, around the wrist. The range of motion was normal, but cross-fluctuation was present. There was no sensory deficit; median nerve involvement was ruled out and distal pulses were intact. The patient had no known personal or family history of tuberculosis. Routine blood investigations were unremarkable. His ESR was 25 mm/hr, and chest radiograph was normal.

**Figure 1:**
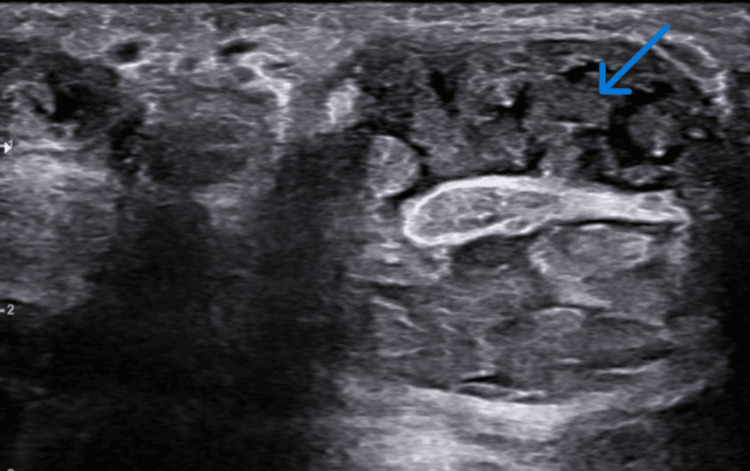
Ultrasound scan demonstrating multiple, slightly echogenic nodules with intervening fluid at the volar aspect of the hand

On ultrasound (Figure 1), fluid and multiple mildly echogenic nodular corpuscles were noted in the common flexor tendon sheath, and radial and ulnar bursa with a mildly thickened wall extending from the distal forearm to the mid palm. No abnormal vascularity was noted.

**Figure 2:**
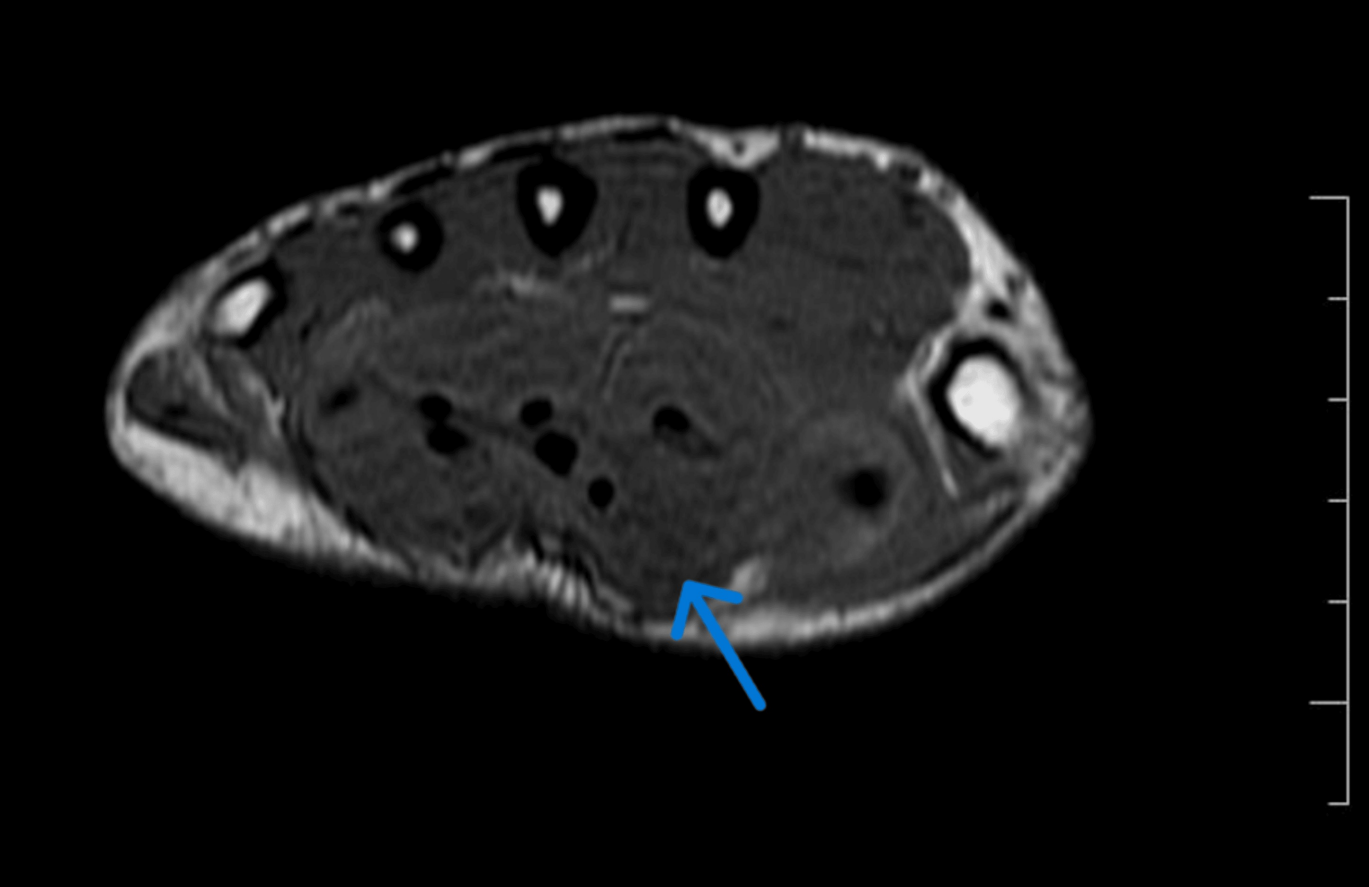
Axial T1-weighted MR image distal to the carpal tunnel demonstrates distended radial and ulnar bursae with numerous isointense corpuscles (rice bodies)

**Figure 3:**
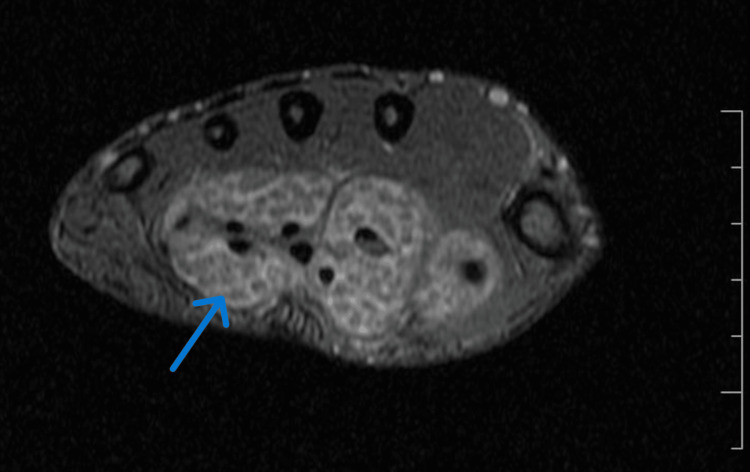
Axial T2* GRE MR image of the hand showing distended radial and ulnar bursae with hyperintense fluid and numerous rice bodies with no blooming artifact GRE: gradient echo

**Figure 4:**
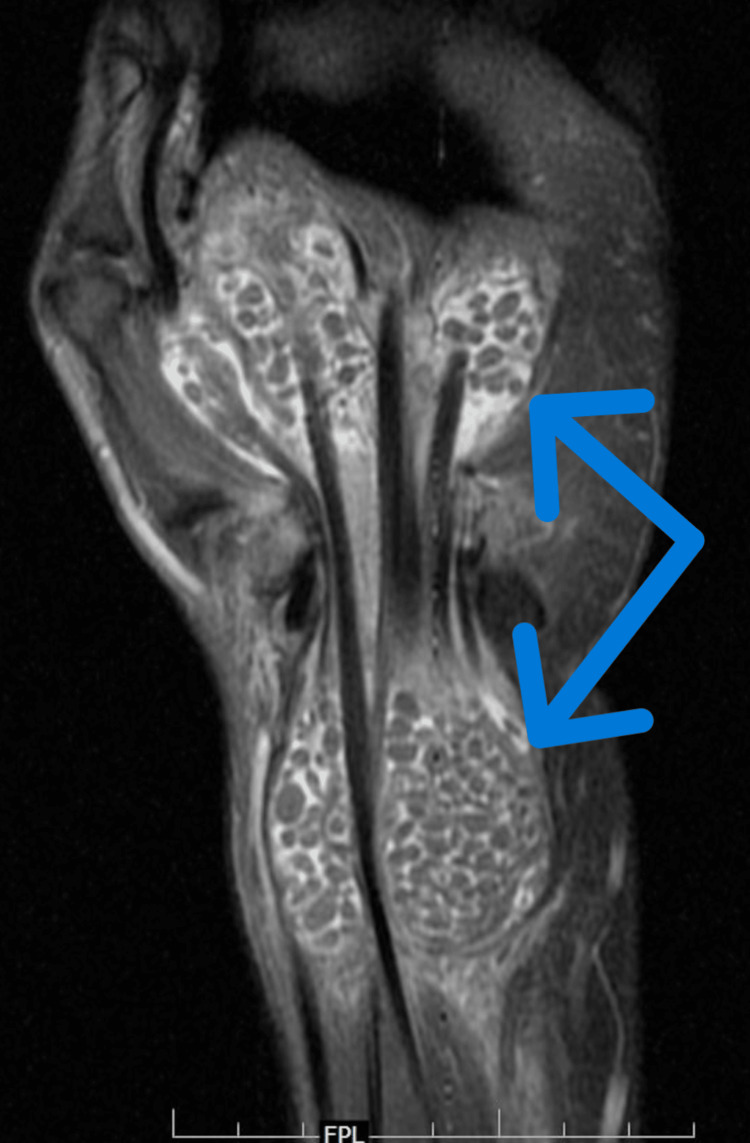
Coronal PDFS MR image showing the hourglass-shaped collection across the carpal tunnel extending from the distal forearm to the level of metacarpal shafts with numerous rice bodies PDFS: proton density fat-saturated

On MRI (Figures 2-4), mild synovial thickening with an hourglass-shaped collection was noted along the flexor digitorum and flexor pollicis tendons, extending proximal and distal to the carpal tunnel, from the distal forearm to the level of metacarpal shafts, with extension to the flexor pollicis longus tendon sheath distally. The collection was studded with numerous T1, T2 and proton density fat-saturated (PDFS) isointense nodules. Findings were compatible with flexor tenosynovitis with fluid and rice bodies. There was no involvement of underlying bones.

**Figure 5:**
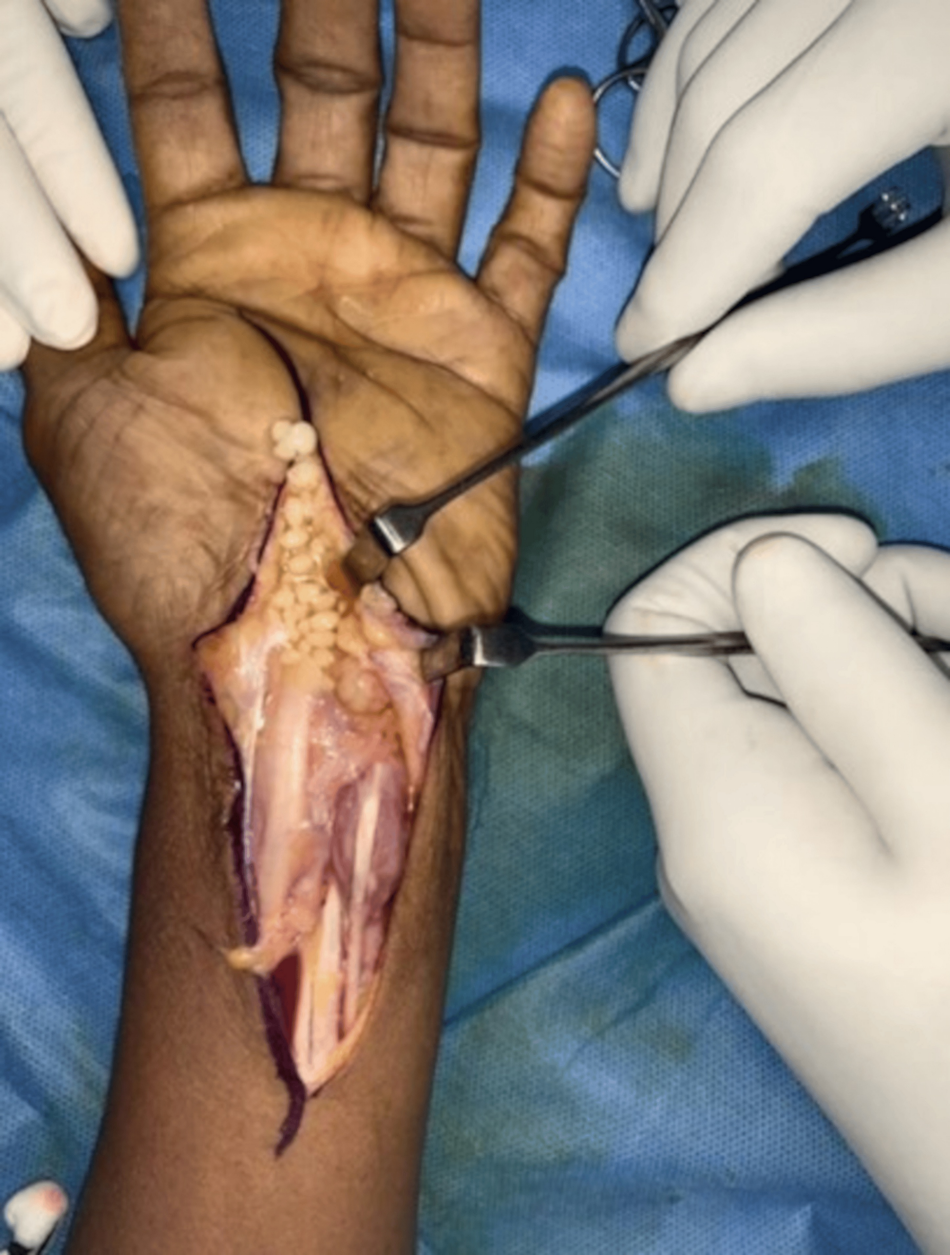
Intraoperative photograph showing synovial fluid and multiple rice bodies around flexor tendons

An open excisional biopsy of the lesion was performed under regional anesthesia. A lobulated, encapsulated synovial mass was identified and the flexor retinaculum was released. Thickened synovium, glistening white rice bodies and synovial fluid were noted encasing all flexor tendons in the distal forearm and hand (Figure 5). Rice bodies and thickened synovium were excised.

**Figure 6:**
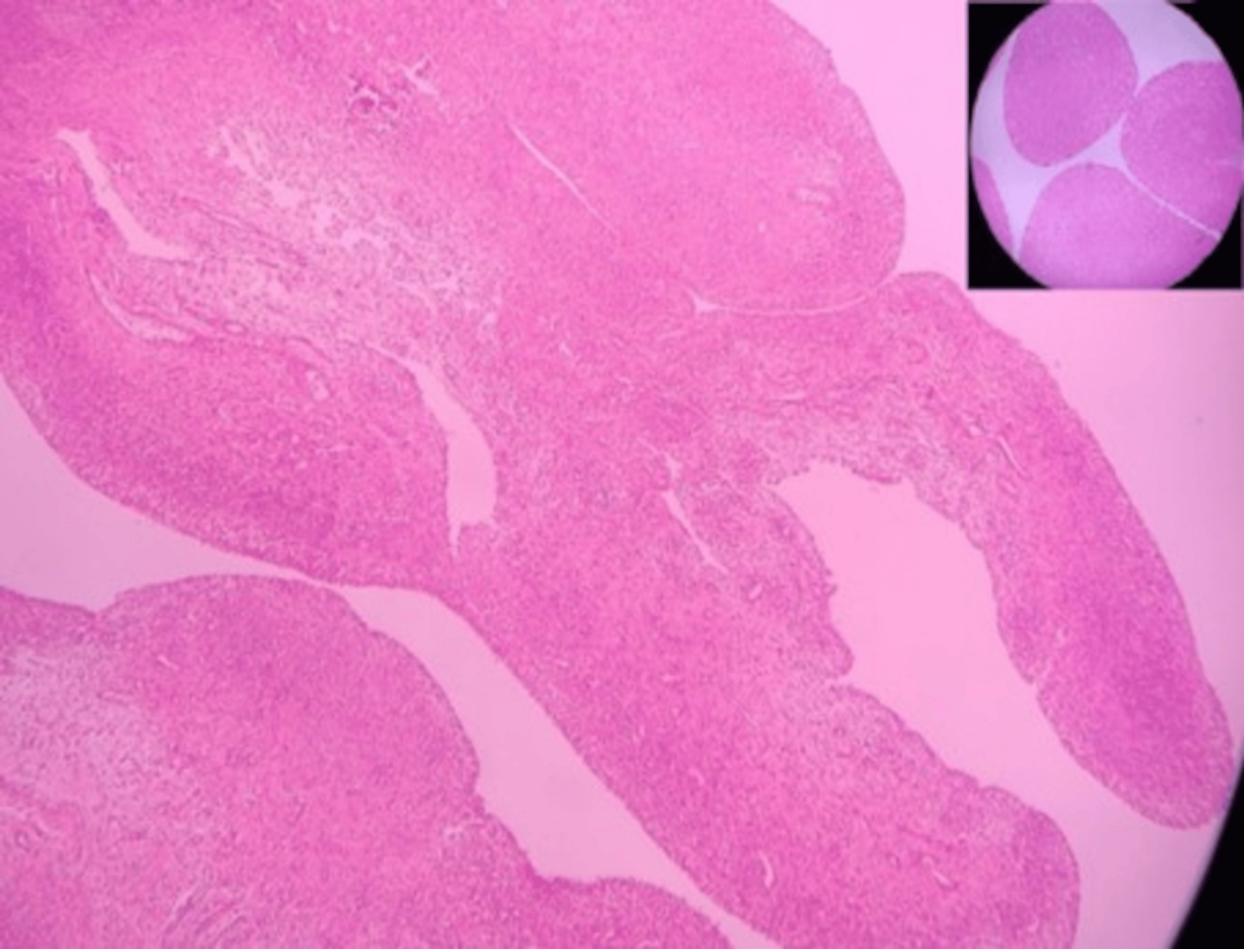
Microscopic image demonstrating hyperplastic synovium (inset: section through rice bodies)

**Figure 7:**
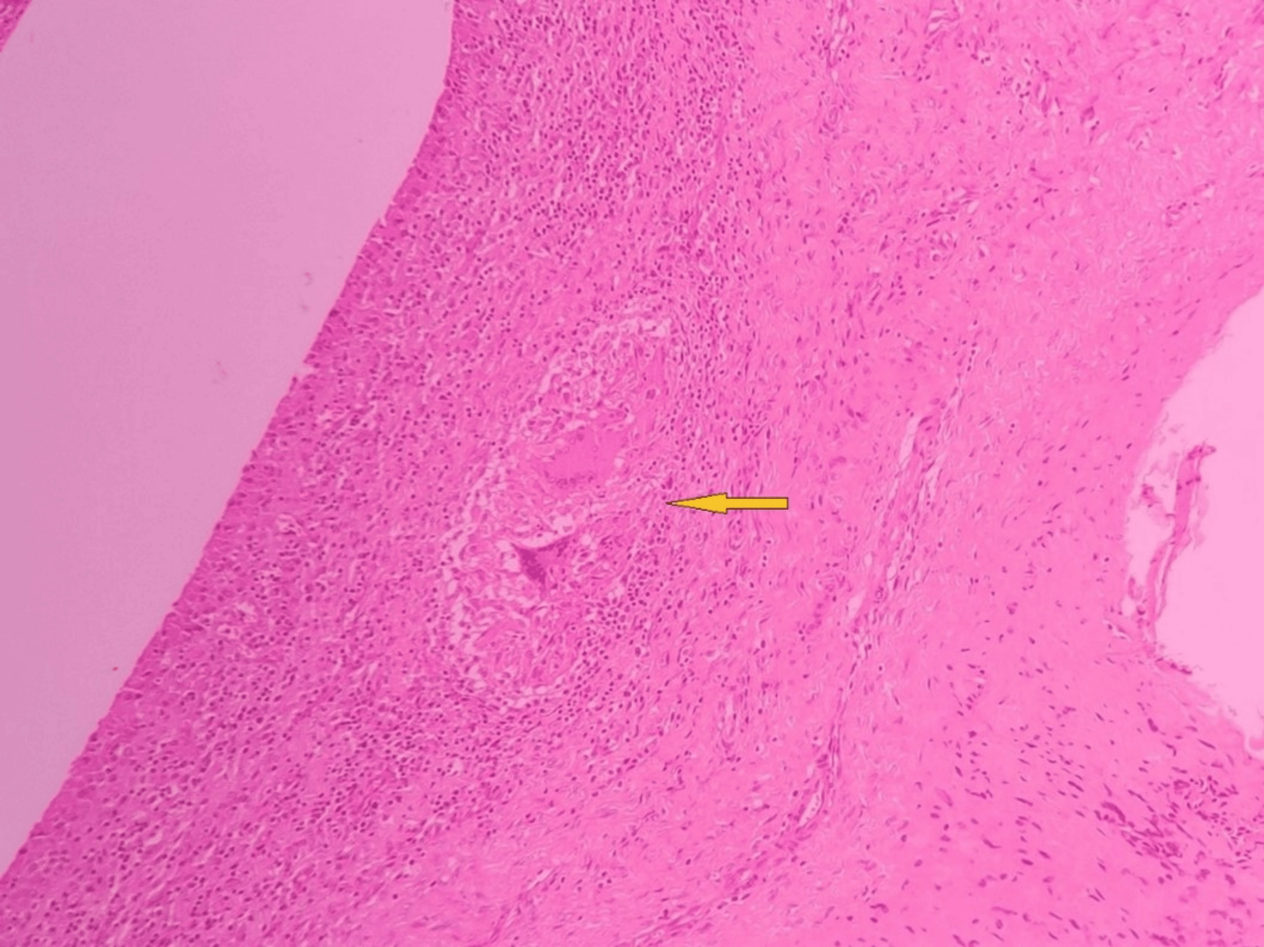
Synovium showing dense mixed inflammation and granuloma (arrow)

**Figure 8:**
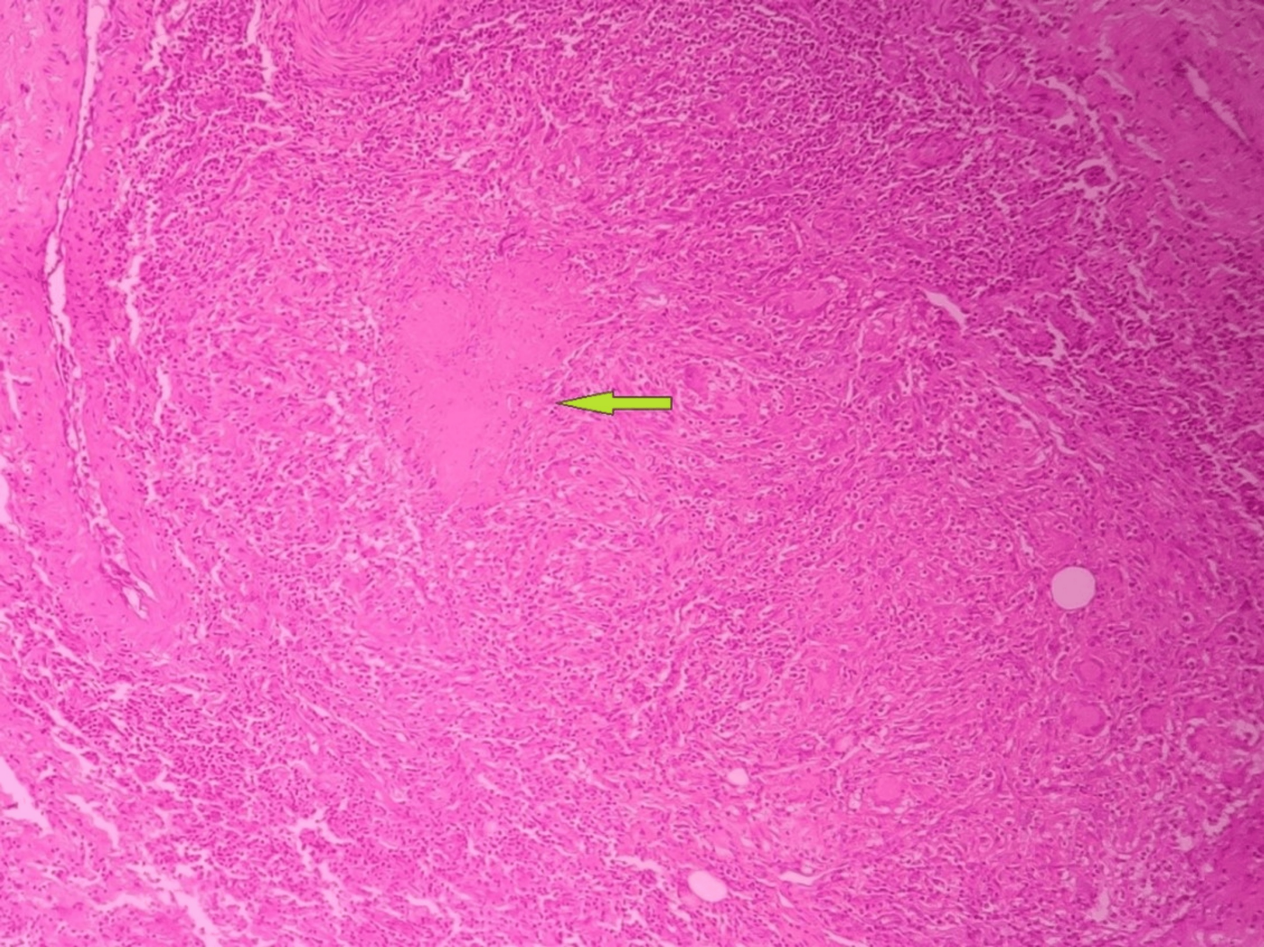
Granuloma with caseation (arrow)

A microscopic examination (Figures 6-8) revealed fibrous tissue with a hyperplastic synovial lining with lymphohistiocytic infiltration. Caseating granulomas consisting of epithelioid cells and Langhans type giant cells were noted. The histopathological examination of rice bodies showed hyalinized sclerotic tissue with a few benign spindle cells. Findings were indicative of chronic granulomatous synovitis of a suggested tuberculous etiology. Acid-fast bacilli (AFB) stain results were negative.

Based on imaging and histopathological findings, anti-tubercular therapy was administered for nine months. On follow-up, the patient was been asymptomatic with no recurrence.

## Discussion

Tuberculous flexor tenosynovitis is a rare and slowly progressing disease that manifests with non-specific symptoms like pain and swelling of the wrist, usually with lack of systemic symptoms, delaying the diagnosis, as in the present case. Paresthesia may occur due to median nerve compression. Disease spread can occur via direct inoculation or hematogenous route [2]. A remote history of trauma was present in our case, which could be a possible mechanism of acquiring the disease.

On the flexor aspect, the synovial sheaths comprise the two palmar bursae: the ulnar and the radial bursae that envelop the tendons traversing the carpal tunnel. These bursae begin proximally near the distal margin of the pronator quadratus and extend up to the mid palm. The radial bursa envelops the flexor pollicis longus tendon and communicates with its sheath in 100% of cases, as reported in various studies [3-5]. The ulnar bursa surrounds the eight flexor digitorum tendons by means of three invaginations - deep, middle and superficial [3]. The ulnar bursa communicates with the flexor tendon sheath of the little finger in approximately 50%-80% of individuals [3,5]. In cases of infection, spread across flexor tendon sheaths of the thumb and little finger via the palmar bursae can result in the classic horseshoe abscess; proximal extension into the forearm via the carpal tunnel may also occur, as evident in present case. Usually, the ulnar bursa does not communicate with the second, third, and fourth digital flexor tendon sheaths; it terminates via short projections at the level of the metacarpals. The communication between the ulnar and radial bursae at the level of the carpal tunnel is common, occurring in 50%-100% of individuals, as reported [3,5]. An ‘intermediate bursa’ behind the second flexor digitorum tendon has been identified as a site of communication [3,5]. The Space of Parona is a potential space in the distal forearm between the pronator quadratus fascia and flexor digitorum profundus tendon sheath. It is not in direct continuity with the palmar bursae, and infection of this space can occur via rupture of these bursae [6].

Rice bodies are composed of fibrin and form due to chronic synovial inflammation [7,8]. They can occur in many chronic disorders like rheumatoid arthritis, systemic lupus erythematosus, sarcoidosis, tuberculosis, and atypical mycobacterial infection [9].

Imaging plays an important role in diagnosing chronic flexor tenosynovitis. Radiographs may show soft tissue masses or presence of bone destruction. CT can show the nature and extent of the lesion and bony involvement. Ultrasound is useful in assessing the nature and extent of the lesion, relationship with flexor tendons, synovial thickening, and median nerve compression. A heterogeneous cystic lesion encasing the flexor tendons of the hand proximal and distal to the flexor retinaculum is usually seen. Rice bodies appear as multiple small isoechoic to slightly echogenic nodules within the lesion. When very small, rice bodies may appear as a soft tissue mass, debris, or viscous fluid in the distended bursa. MRI is an excellent modality for imaging suspected cases of chronic flexor tenosynovitis as it provides better characterization of the lesion. It demonstrates the involvement of tendon sheaths, radial or ulnar bursa, effusion, synovial thickening, and rice bodies. Rice bodies usually appear isointense on T1-weighted (T1W) MRI and iso- to hypointense on T2-weighted (T2W) MRI. Thickened synovium appears hypointense. The lesion may show rim enhancement on a post-contrast study. In our case, rice bodies appeared isointense on both T1W and T2W MRI. Post-contrast imaging was not done due to financial constraints.

The differential diagnosis on MRI includes synovial chondromatosis and tenosynovial giant cell tumor. In synovial chondromatosis, there is chondroid metaplasia of the synovium with formation of multiple intra-articular chondral bodies that usually have a high T2 signal. When calcified, they show a low signal on T2 and gradient echo (GRE) images and can be identified on plain radiographs aiding in the diagnosis. A tenosynovial giant cell tumor is a benign neoplastic lesion that arises from the tendon sheath. It appears as soft tissue nodules that are hypointense on T1W and T2W MRI, with 'blooming' on GRE due to the presence of hemosiderin. Also, it presents with contrast enhancement.

In the present case, ultrasound and MRI findings clearly depicted the presence of rice bodies, effusion, and synovial thickening. Histopathologic examination shows epithelioid granulomas with multinucleate giant cells and caseous necrosis. Synovial fluid AFB staining for mycobacteria may not always yield a positive result [10], as in our case. Tissue culture is the most important test to identify mycobacteria, but it can take weeks to months. Therefore, it becomes important to identify the disease based on clinical features, imaging, and histopathology, and start empiric anti-tubercular therapy.

## Conclusions

Tuberculosis should be suspected in patients having chronic tenosynovitis. Rice bodies are an uncommon manifestation of chronic synovial inflammation and have characteristic imaging and histological features. Early diagnosis and treatment of the condition can prevent complications. Synovectomy and anti-tubercular therapy are the mainstay of treatment for chronic tubercular tenosynovitis.
